# Plasma pTau217 as a Strong Predictor of Alzheimer's Disease in the Korean Population

**DOI:** 10.1002/alz70856_102515

**Published:** 2025-12-25

**Authors:** Sarang Kang, Kyu Yeong Choi, Gyungah R Jun, Kun Ho Lee

**Affiliations:** ^1^ Gwangju Alzheimer's and Related Dementia (GARD) Cohort Research Center, Chosun University, Gwangju, Korea, Republic of (South); ^2^ Kolab Inc., Gwangju, Korea, Republic of (South); ^3^ Department of Biostatistics, Boston University School of Public Health, Boston, MA, USA; ^4^ Boston University School of Public Health, Boston, MA, USA; ^5^ Boston University Chobanian & Avedisian School of Medicine, Boston, MA, USA; ^6^ Department of Ophthalmology, Boston University Chobanian & Avedisian School of Medicine, Boston, MA, USA; ^7^ Biomedical Genetics Section, Department of Medicine, Boston University Chobanian & Avedisian School of Medicine, Boston, MA, USA; ^8^ Alzheimer's Disease Research Center, Boston University Chobanian & Avedisian School of Medicine, Boston, MA, USA; ^9^ Eisai Andover Innovative Medicines (AiM) Institute, Andover, MA, USA; ^10^ Korea Brain Research Institute, Daegu, Korea, Republic of (South); ^11^ Biomedical Science, Chosun University, Gwangju, Gwangju, Korea, Republic of (South)

## Abstract

**Background:**

Plasma biomarkers offer a non‐invasive and accessible method for the early detection of Alzheimer's disease (AD). While plasma phosphorylated tau 217 (pTau217) has shown promise in European populations, its predictive capacity in Koreans is less explored. This study examined the diagnostic potential of pTau217, glial fibrillary acidic protein (GFAP), and neurofilament light chain (NfL) in the Gwangju Alzheimer's & Related Dementia (GARD) cohort, a large‐scale dementia cohort in Korea.

**Method:**

We measured plasma levels of pTau217, GFAP, and NfL in 725 age‐matched participants including 442 cognitively normal (CN), 224 mild cognitive impairment, and 59 AD‐related dementia (ADRD). Biomarkers were quantified using single‐molecule array (Simoa) assays, and amyloid positivity was determined through PET imaging. Neuropsychological assessments, APOE genotyping, and pathological analyses were also conducted.

**Result:**

We observed no association between age at measurement or sex across CN, MCI, and ADRD groups. The levels of pTau217, GFAP, and NfL were significantly associated with AD dementia and amyloid PET positivity (*p* <0.05), while the presence of the APOE e4 allele was not significant for AD dementia in this dataset (*p* = 0.193). Among these plasma biomarkers, pTau217 demonstrated the strongest predictive value for amyloid positivity, with an area under curve (AUC) of 0.935, compared to GFAP (AUC=0.822) and NfL (AUC=0.635). While pTau217 had a lower predictive value for AD status (AUC=0.778), it is still outperformed GFAP (AUC=0.706) and NfL (AUC=0.674). Additionally, pTau217 exhibited a strong correlation with AD progression, particularly in amyloid‐positive individuals, with steeper age‐related increases. In addition to pTau217, we discovered and confirmed the potential of novel blood markers to predict neurodegeneration in amyloid‐negative individuals.

**Conclusion:**

This study confirmed the potential of plasma biomarkers, particularly pTau217, in predicting amyloid positivity and AD progression in the Korean population. Plasma pTau217 emerged as the most robust plasma biomarker for detecting amyloid positivity, outperforming GFAP and NfL. These findings suggest that pTau217 may serve as a reliable cross‐ancestry plasma biomarker for predicting the early development of amyloid positivity before onset of cognitive symptoms.

